# Expanding the family of genetically encoded voltage indicators with a candidate Heliorhodopsin exhibiting near-infrared fluorescence

**DOI:** 10.1016/j.jbc.2023.104771

**Published:** 2023-04-29

**Authors:** Srividya Ganapathy, Xin Meng, Delizzia Mossel, Mels Jagt, Daan Brinks

**Affiliations:** 1 Department of Imaging Physics, Delft University of Technology, Delft, The Netherlands; 2 Department of Pediatrics & Cellular and Molecular Medicine, UCSD School of Medicine, San Diego, USA; 3 Department of Molecular Genetics, Erasmus University Medical Center, Rotterdam, The Netherlands

**Keywords:** voltage sensors, neuroscience, fluorescence microscopy, rhodopsins, protein engineering

## Abstract

Genetically encoded voltage indicators, particularly those based on microbial rhodopsins, are gaining traction in neuroscience as fluorescent sensors for imaging voltage dynamics with high-spatiotemporal precision. Here we establish a novel genetically encoded voltage indicator candidate based on the recently discovered subfamily of the microbial rhodopsin clade, termed heliorhodopsins. We discovered that upon excitation at 530 to 560 nm, wildtype heliorhodopsin exhibits near-infrared fluorescence, which is sensitive to membrane voltage. We characterized the fluorescence brightness, photostability, voltage sensitivity, and kinetics of wildtype heliorhodopsin in HEK293T cells and further examined the impact of mutating key residues near the retinal chromophore. The S237A mutation significantly improved the fluorescence response of heliorhodopsin by 76% providing a highly promising starting point for further protein evolution.

Detailed studies of neural circuitry and computation are contingent upon resolving the electrical dynamics of several neurons in parallel with high spatiotemporal precision. Direct visualization of changes in neural membrane potential has been facilitated by engineering bright and sensitive probes of which the fluorescence is modulated by changes in membrane voltage. These engineered transmembrane proteins are termed genetically encoded voltage indicators (GEVIs) (1). Various GEVI families have been optimized over the past years, and particularly GEVIs based on microbial rhodopsin proton pumps have enabled the recording of activity in an ensemble of neurons with submillisecond response time (2).

The first rhodopsin-based GEVI was derived from the bacterial Proteorhodopsin, discovered due to the success of metagenomic sequencing efforts in Monterey Bay (3). Another proton pump, Archaerhodopsin-3 (Arch) from the archaea *Halorubrum sodomense,* was found to be a better GEVI candidate for expression in mammalian cells (4). The first Arch versions were very dim and required several iterations of molecular evolution (4, 5, 6, 7, 8). Many flavors of Arch-based GEVIs have since been developed with improved brightness, sensitivity, and membrane targeting, the most recent ones being Archon1 and Quasar6 (4, 9, 10). Quasar6a has a reported voltage sensitivity of 73% ± 8% per 100 mV in human embryonic kidney (HEK293T) cells and a significant improvement in the signal to noise ratio (SNR) in neurons over earlier versions (10). The evolved brightness of Archon1 and Quasar6a has enabled *in vivo* imaging in mice and zebrafish, in combination with a spectrally orthogonal Channelrhodopsin for *in vivo* all-optical electrophysiology (9, 10, 11).

Arch-based GEVIs exhibit complex photophysics, and various models have been proposed over time to shed light on its voltage sensitivity (12, 13). Wildtype Arch and some other rhodopsins typically display weak fluorescence arising from the retinal chromophore (14). Retinal is covalently bound to the protein *via* a Schiff-base linkage with a Lysine, which is normally protonated. The near-infrared fluorescence of this retinal protonated Schiff base (RPSB) is modulated by the charge distribution of nearby residues lining the binding pocket. Light absorption initiates the photocycle of the protein *via* a sequence of conformational changes, which in turn can impact RPSB fluorescence due to changes in electrostatic interactions. Canonically, photon absorption in the ground state leads to isomerization of the RPSB from *all-trans* to 13-*cis* and relocation of its proton to a negatively charged counterion acceptor (M-state). Photophysical characterization of Arch suggests that the reprotonation of the Schiff base (M→N) is influenced by membrane voltage and populates the N-state, where an increased likelihood of photon absorption leads to a fluorescent Q-intermediate (12).

The complex photophysics of Arch and the high tunability of its fluorescent brightness, voltage sensitivity, and kinetics by targeted mutations have made it an exciting candidate to investigate and evolve further as a GEVI (4, 7, 9, 15, 16). However, besides Arch only a handful of rhodopsin proton pumps have been engineered as GEVIs, despite the expansive diversity of the microbial rhodopsin family. Other rhodopsins with different ionic transport or sensory functions remain vastly unexplored as potential GEVIs, despite all having the same tunable retinal chromophore in common. In addition, novel rhodopsins with unique properties are continuously being added to the family, which deserve further exploration of their bioengineering potential. Recently, metagenomic sequencing in Lake Kinneret led to the discovery of a new family of rhodopsins termed Heliorhodopsins (17). They were found to be abundant in the photic zone occurring in diverse host species ranging from bacteria to viruses (17).

Heliorhodopsins are also heptahelical retinal binding proteins, but they are remarkably different from other microbial rhodopsins due to an inverted insertion in the membrane with a cytoplasmic N terminus (17). No clear ion translocation has been found, with the exception of a viral heliorhodopsin, which functions as a light-gated proton channel (18). Heliorhodopsins display a relatively long photocycle (∼1–5 s) (17, 19) indicating that they may have some kind of sensory or signaling role. Their precise physiological functions are thought to be diverse and are mostly unknown. However, very recent evidence from bacterial heliorhodopsins allude toward a major role as regulators of enzymatic activity for processes such as nitrogen assimilation (20) and DNA repair (21). Other studies have also shown an influence on membrane signaling *via* light-induced lipid remodeling (22) or the transport of membrane-impermeable molecules (23). The crystal structures of two heliorhodopsin variants have recently been resolved, shedding some light on their unusual properties (19, 24). Bacterial HeR-48C12 contains a large cytoplasmic RPSB cavity with several polar residues and water molecules. This arrangement enables transient proton transfer from the RPSB and back, *via* a proton-accepting group (PAG) involving H23 and H80. This polar H-bonded environment is highly amenable for tuning the spectral properties of retinal (25), making Helios an interesting candidate for bioengineering.

In this study we demonstrate the potential of Heliorhodopsin (bacterial HeR-48C12, Fig. 1*A*) to function as a fluorescent indicator of membrane voltage. We show that wildtype Heliorhodopsin displays voltage-dependent fluorescence, which can be improved with targeted mutations in the retinal-binding pocket. This research paves the way for further evolution of Heliorhodopsin-based GEVIs and opens the door for engineering other members of the microbial rhodopsin clade.

**Figure 1:**
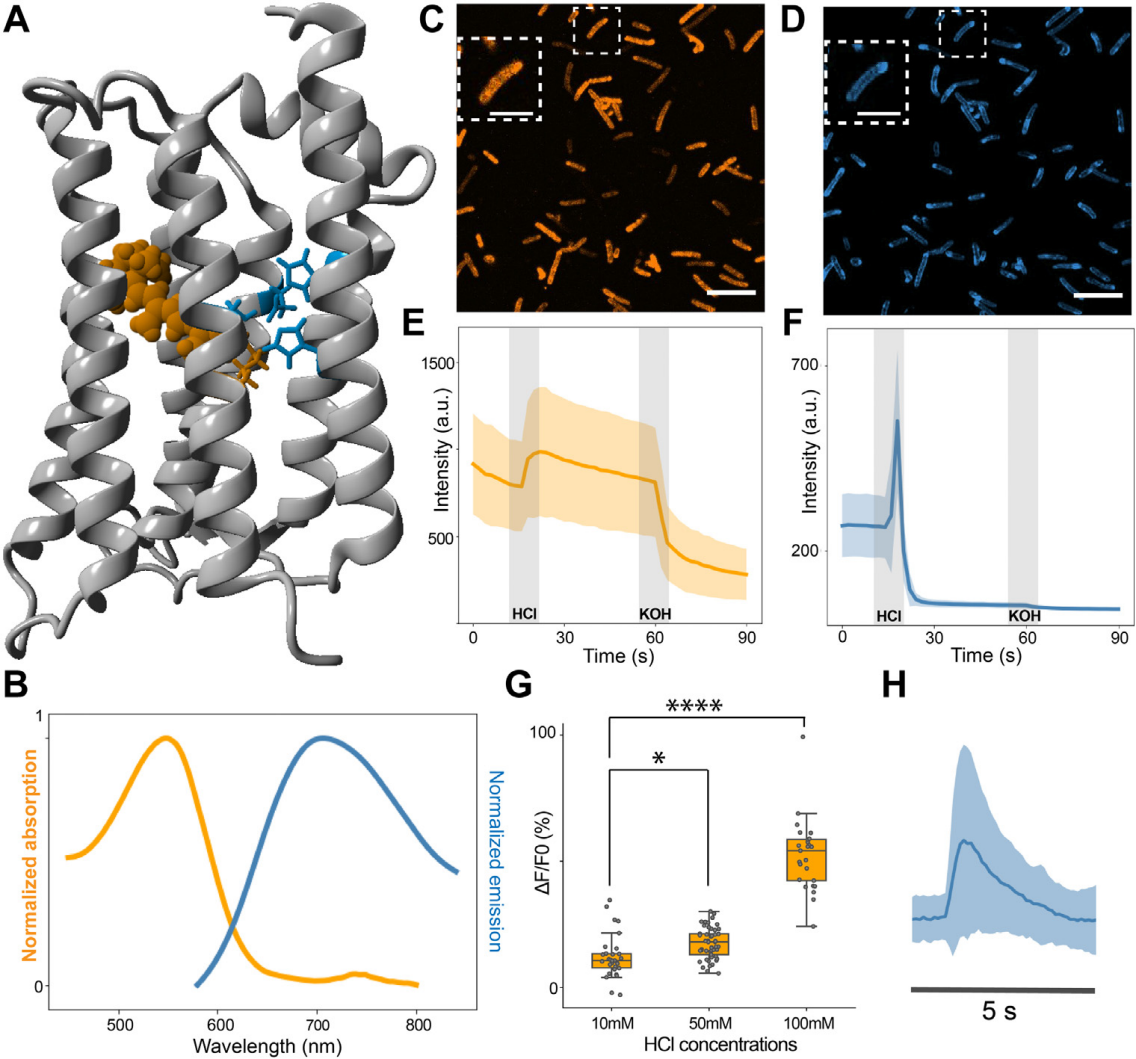
**Preliminary characterization of Helios fluorescence.***A*, crystal structure of Helios48C12 (PDB 6su3) displaying the retinal Schiff base in *orange* and residues involved in color tuning in *blue*. *B*, normalized absorption and emission spectra of purified WT Helios. *C* and *D*, representative confocal fluorescence images of *E. coli* expressing Helios under 561 nm (*C*) and 640 nm (*D*) illumination, with the inset representing a zoom-in of an individual cell. The scale bars represent 10 μm; in insets, 5 μm. *E* and *F*, fluorescence response of *E. coli* expressing Helios to 25 mM HCl and 25 mM KOH addition under 561 nm (*E*) and 640 nm (*F*) illumination (n = 45 cells). Videos were recorded at 1 fps. The *thick line* is the mean response with the lighter region representing the SD; the *gray blocks* indicate the time point of HCl or KOH addition. *G*, quantification of *E. coli* fluorescence response under 561 nm illumination to increasing concentrations of extracellular HCl addition (10 mM:12.23 ± 8.52; n = 35; 50 mM: 17.55 ± 6.13; n = 50; 100 mM: 52.28 ± 14.08; n = 39). All statistics are mean ± SD; in the boxplots the boundaries of the whiskers are based on an interquartile range of 1.5, each *gray dot* in the boxplot represents a cell. The *p*-value of the one-way ANOVA test is 1.16e-30. The *p*-values of 50 mM and 100 mM against 10 mM are 0.045 and 1.90e-14, Tukey’s post hoc test. *H*, Spinning-disk confocal fluorescence response of Helios at 640 nm illumination recorded at 10 fps. All statistics are mean ± SD.

## Results and discussion

### Expression and fluorescence imaging of Helios in *E. coli*

In order to assess the fluorescence properties of wildtype Heliorhodopsin (hereby called Helios), we did a preliminary characterization in *Escherichia coli*. Recombinant N-terminal 6XHis-tagged Helios was overexpressed in *E. coli* and purified using Ni^2+^ NTA affinity chromatography. The absorption spectrum of purified Helios displayed a λ_max_ at 549 ± 1 nm, in good agreement with reported values (17). Upon excitation at 550 nm, we obtained a distinct emission band extending from 600 nm into the near-infrared region, peaking at ∼700 nm (Fig. 1*B*). Based on comparison of absorbance and emission integral to fluorophores with known quantum yield, we estimate a fluorescence quantum yield of 6 × 10^−4^, which is in agreement with the typical range reported for other microbial rhodopsins (4, 5, 26).

Next, we directly imaged Helios in intact *E. coli* cells using confocal microscopy. Bright fluorescence was seen localized to the cell membrane when imaged under 561 and 640 nm illumination (Fig. 1, *C* and *D*), although with some moderate photobleaching (Fig. S1). Biexponential fitting of the photobleaching response yielded time constants of 20 and 120 s for the 561 and 640 nm fluorescence, respectively (Fig. S1). We tested the sensitivity of this fluorescence to changes in extracellular pH, as a preliminary indicator of voltage sensitivity. Upon addition of 25 mM HCl to the cells, an increase in fluorescence was seen in the 561 nm channel well above the photobleaching background (Fig. 1*E*). This step response in fluorescence was roughly linear with increasing concentrations of HCl (Fig. 1*F*) and could be reversed upon addition of 25 mM KOH (Fig. 1*E*). However, in the 640 nm channel, we observed an initial large rise in fluorescence followed by rapid quenching of the signal within 3 to 4 s (Fig. 1, *G* and *H*). This fluorescence could not be recovered with dark incubation or KOH addition (Fig. 1*G*). Furthermore, it was not impacted by the presence or order of the 561-nm illumination pulse. This quenching reaction possibly involves a complex photocycle pathway characterized by the pH-dependent inactivation of a near-infrared photointermediate. Since our interest is in the use of Heliorhodopsin as GEVI, which requires a linear and reversible response to membrane voltage, we focused on the ∼561 nm fluorescence of Helios.

### Characterization of Helios WT in HEK293T

**Figure 2:**
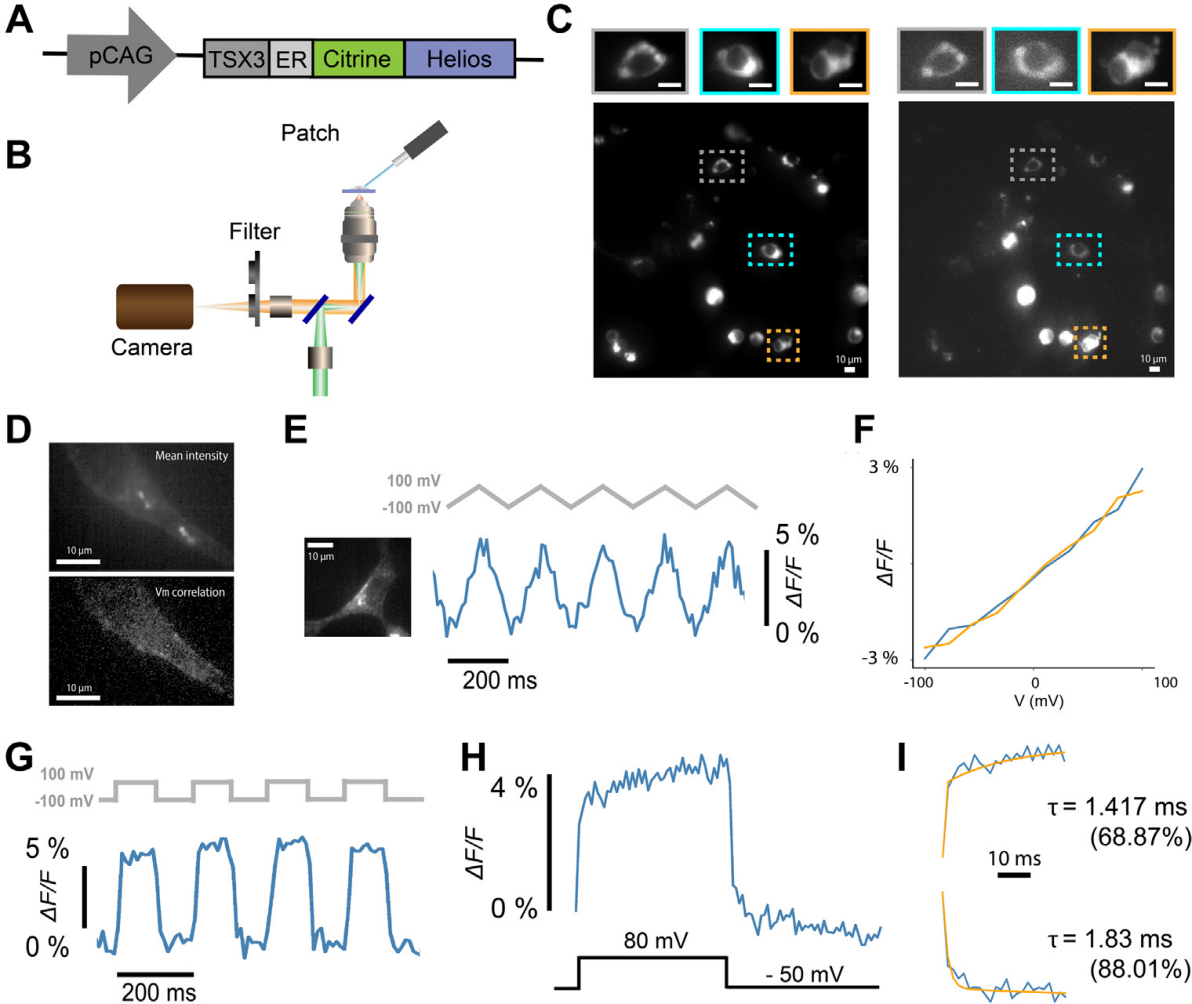
**Characterization of voltage sensitivity of WT Helios in HEK293T cells.***A*, schematic of the plasmid for expression of Helios under the pCAG promoter with the targeting motifs (TSX3, ER) and Citrine as a fusion protein. *B*, an illustration of the setup used for simultaneous fluorescence imaging with voltage clamp electrophysiology. *C*, full field of view fluorescence images of HEK293T cells expressing Helios WT under 488 nm (*left*) and 532 nm excitation (*right*). The zoom-in view on the *top* displays representative individual cells. The scale bars represent 10 μm. *D*, *top*: mean intensity image from a video of a voltage-clamped cell expressing Helios. *Bottom*: The correlation map between the video and the membrane voltage. *E*, characterization of the fluorescence response of Helios to whole cell voltage clamp. *Left*: Fluorescence image of the patched HEK293T cell. *Right*: Helios fluorescence response to 200 mV voltage ramps recorded at 100 fps. The illumination intensity was 87.6 mW/mm^2^. *F*, averaged upswing and downswing traces from 25 trials. *G*, Helios fluorescence response to 200-mV voltage steps recorded at 100 fps. *H*, averaged fluorescence response to 130-mV voltage steps recorded at 500 fps. *I*, biexponential fitting analysis and kinetics of voltage-sensitive fluorescence.

Helios was cloned into an expression vector for HEK293T cells driven by the strong pCAG promoter. Based on prior efforts to optimize the membrane trafficking of Arch (4), we added a trirepeat of targeting sequences and endoplasmic reticulum motif (TSX3ER2) with Citrine as a fusion protein for localization (Fig. 2*A*), based on the design of Quasar3 (27). HEK293T expressing pCAG-Helios was imaged using a home-built epifluorescence microscope with a patch clamp add on for electrophysiology (Fig. 2*B*). Strong near-infrared fluorescence (660–800 nm) could be seen when imaged at 488 and 532 nm in agreement with the measurements in *E. coli* (Fig. 2*C*). Interestingly, no fluorescence was seen upon 639-nm excitation, possibly due to differences in binding-pocket conformation in the mammalian expression system *versus E. coli*. While Helios expression was mostly localized to the plasma membrane (Fig. 2*C*), intracellular aggregates and overexpression leading to cell death were also seen, likely due to the strong pCAG promoter. The ratios of membrane fluorescence to soma fluorescence, as measured by quantifying the image intensity at the cell contour and soma, respectively, show similar values around 1 for WT and mutants, indicating that expression is distributed within the cell (Fig. S3). Helios exhibited moderate photobleaching at 532 nm (Fig. S2), with a fast constant of 11.16 ± 3.39 ms (54.9% ± 0.14%) and a slow constant of 158.39 ± 3.39 ms (n = 6 cells; all statistics are mean ± standard deviation [SD]).

We assessed the voltage sensitivity of Helios at room temperature by modulating the membrane potential of HEK293T cells expressing Helios using whole cell patch clamp electrophysiology and measuring the changes in fluorescence. Correlation of per-pixel fluorescence change with the change in membrane voltage showed characteristic localization of the voltage-sensitive fluorescence at the cell membrane (Fig. 2*D*). In combination with mean intensity images displaying significant somatic fluorescence (Fig. 2, *C* and *D*), this indicates Helios displays proper membrane trafficking but is overexpressed in most cells. Voltage ramps of 200 mV were used at a frequency of 5 Hz (Fig. 2*E*). The concurrent fluorescence response was recorded at 532 nm on a sCMOS camera at a frame rate of 100 Hz. Helios displayed a linear response to membrane voltage over a −100 to +100 mV range (Fig. 2*F*). Subsequently, 200-mV voltage pulses were delivered at a frequency of 5 Hz, for a total duration of 5 s. Here, the 532 nm fluorescence response was recorded at a frame rate of 100 Hz (Fig. 2*G*) or 500 to 1000 Hz for high-speed characterization of the time constants (Fig. 2, *H* and *I*). The signal was temporally averaged after subtracting the background signal and correcting for photobleaching. The fractional change in fluorescence was extracted and normalized to a 200-mV step (from −100 mV to +100 mV) yielding a ΔF/F_0_ of 6.14% ± 1.35% per 200 mV for Helios WT (n = 7 cells; mean ± SD) Biexponential fitting of the fluorescence trace measured at 500 Hz yielded a fast time constant for the upswing of 2.06 ± 0.47 ms (62%) and of 2.40 ± 0.40 for the downswing (n = 3 cells). (Fig. 2*I*).

### Comparison between Helios mutants

The voltage response of Helios appeared to be substantially dominated by the recording speed in our measurements. We were intrigued by the step-like fluorescence response to the 200-mV voltage block recorded at 500 Hz, since this is a comparable speed at which *in vivo* voltage imaging is typically performed where Arch-based sensors tend to have comparable or slower responses. Therefore, we attempted to improve the fluorescence and voltage sensitivity of Helios by targeted mutations in the retinal-binding pocket.

**Figure 3:**
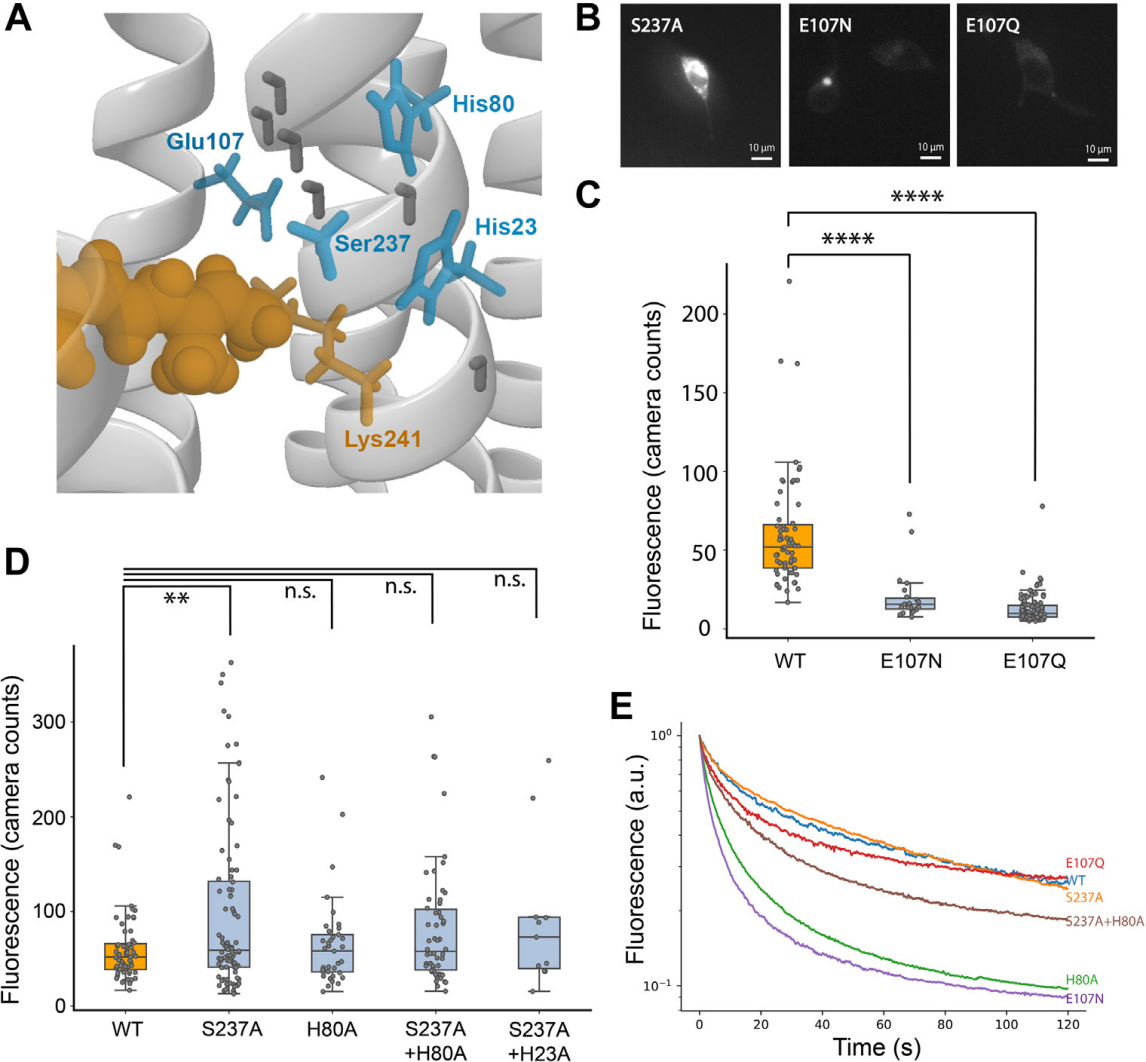
**Brightness and photobleaching of Helios mutants.***A*, binding pocket view of the crystal structure of Helios displaying the retinal Schiff base (*orange*) and key color tuning residues (*blue*). *B*, representative fluorescence images of HEK293T cells expressing the Helios mutants S237A, E107N, and E107Q. The contrast across the images is adjusted to the same level. *C*, comparison of brightness values of HEK293T cells expressing Helios WT (60.6 ± 36.1, n = 63 cells), E107N (20.4 ± 15.9, n = 23 cells), and E107Q 12.7 ± 9.0, n = 107 cells). The *p*-value of the one-way analysis of variance (ANOVA) test is 7.7e-29. The *p*-values of E107N and E107Q against WT Helios both smaller than 1e-8, Tukey’s post hoc test. In the box plots in subfigure (*C*) and (*D*), the boundaries of the whiskers are based on an interquartile range of 1.5, and each gray point in the plot represents one measurement. *D*, comparison of brightness values of HEK293T cells expressing WT Helios (mean value = 60.6, n = 63 cells), S237A (mean value = 97.8 ± 88.3, n = 92 cells), H80A (mean value = 65.3 ± 47.4, n = 37 cells), S237A + H80A (mean value = 81.7 ± 63.9, n = 54 cells), and S237A + H23A (mean value = 91.4 ± 78.2, n = 11 cells). The *p*-value of the one-way ANOVA test is 9.51e-3. The *p*-values of S237A, H80A, S237A + H80A, and S237A + H23A against WT Helios are 7.68e-3, 0.997, 0.446, and 0.632, Tukey’s post hoc test. The illumination intensity was 37.23 mW/mm^2^. *E*, normalized photo bleaching traces of Helios WT and mutants. All statistics are mean ± SD.

Arch and other rhodopsin proton pumps typically have two negatively charged counterion residues functioning as a complex. In contrast, Helios contains a single E107 as the counterion, which is hydrogen bonded to the RPSB along with an uncharged S237 (Fig. 3*A*) (17, 24). E107 has a pKa at 3.7 and is therefore likely to be unprotonated under physiological conditions (17). The counterion influences the charge distribution of the RPSB, and mutations to neutral residues often cause spectral redshifts in many rhodopsins (28). In Arch, the redshifting D95N and D95Q mutations eliminated the photocurrent and improved the voltage sensitivity (7, 15). Thus, we tested the analogous E107N and E107Q mutations in Helios as a first target. However, the E107N and E107Q mutants showed diminished fluorescence (Fig. 3, *B* and *C*) with the WT brightness being three times higher than E107N and five times higher than E107Q (23 E107N and 107 E107Q cells contribute to the mean brightness calculation; 532 nm, 87.6 mW/mm^2^). No clear voltage response could be measured for these mutants.

This may be attributed to the stronger interaction of the Helios RPSB with the surrounding water-dense Schiff base cavity (24). In most microbial rhodopsins, the counterion usually functions as the primary acceptor for proton transfer from the RPSB upon isomerization (29). However, in heliorhodopsins, the Schiff base cavity collectively functions as the primary proton acceptor (17, 19, 24). The hydrogen-bonded network involving charged binding-pocket residues and water molecules participates in transient transfer of the Schiff base proton from and back to the RPSB (17). A recent study identified color tuning mutations in several of these conserved binding-pocket residues, including H23, H80, and S237, which interact directly with the RPSB (25). We therefore turned our attention to these sites, focusing on the mutations that were reported to cause spectral redshifts, *i.e.*, H23A, H80A, and S237A (25) (Fig. 3*A*).

We screened HEK293T cells expressing the above mutants for their fluorescence brightness (532 nm, 37.23 mW/mm^2^). The averaged brightness of S237A is 23% higher than the WT (the mean fluorescence is calculated from 63 WT cells and 92 S237A cells, *p*-value = 0.00186), while there were no significant changes for the other tested mutations (Fig. 3*D*). However, prolonged illumination of the cells revealed differences in photobleaching behavior among the Helios mutants. While S237A and E107Q had photobleaching rates comparable with the WT, H80A and E107N photobleached significantly faster. Bioexponential fitting of the photobleaching curves revealed that the photobleaching is of a different nature in WT compared with the mutants: WT bleaching is characterized by a relatively strong, high, and fast time constant, whereas that of S237A is dominated by a relatively strong, but less high and slow time constant. (Fig. S2). In a combination mutant, S237A saved some of the long-term fluorescence loss of H80A (Fig. 3*E*).

### Voltage sensitivity of Helios S237A mutants

**Figure 4:**
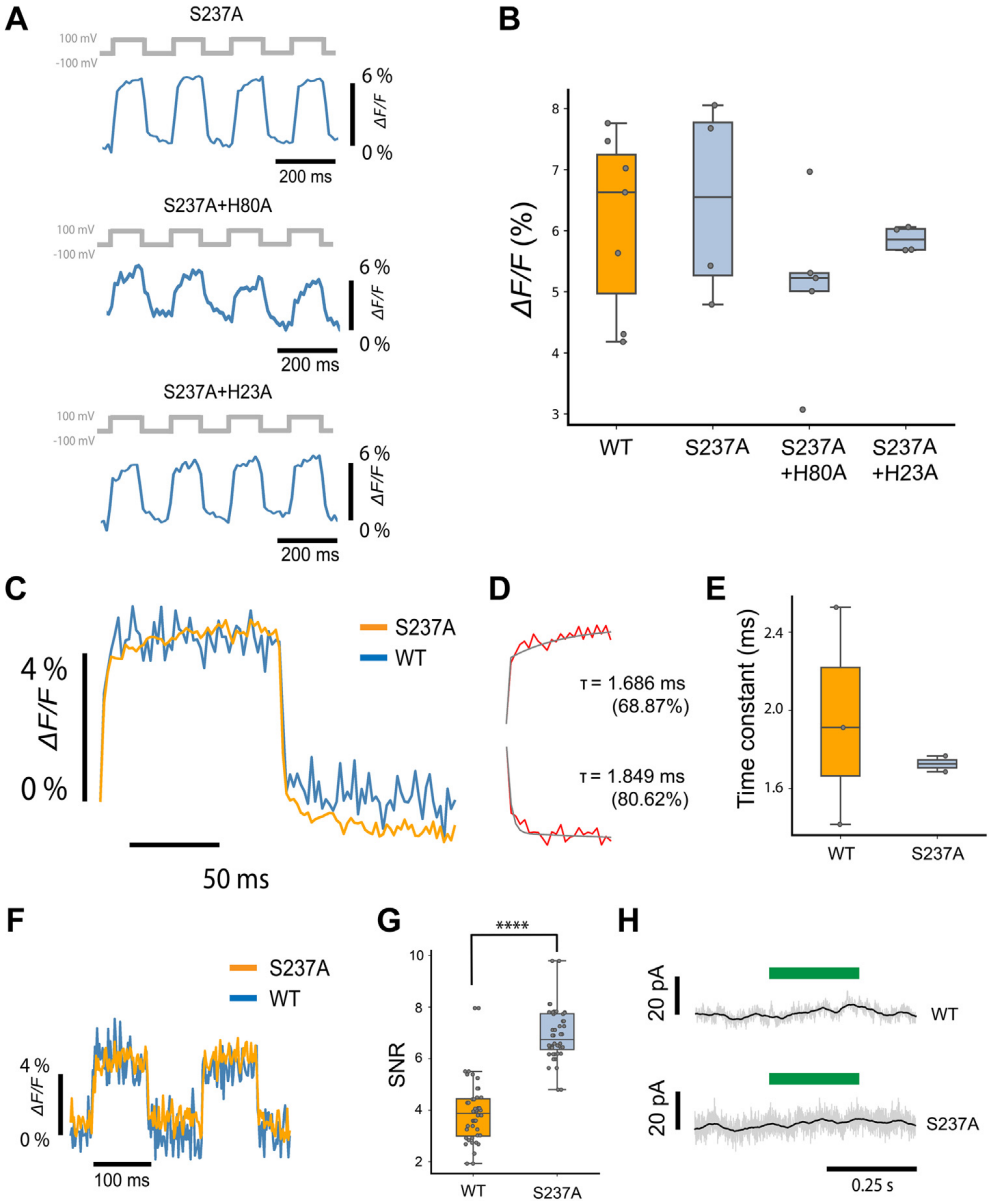
**Characterization of voltage sensitivity of Helios mutants.***A*, fluorescence traces of Helios mutants in response to 200-mV voltage clamp square waves. From *top to bottom*: S237A, S237A + H80A, and S237A + H23A. *B*, comparison of voltage sensitivity between Helios WT and mutants. The voltage sensitivities per 200 mV are Helios WT: 6.14 ± 1.35%; S237A: 6.48 ± 1.40%; S237A + H80A: 5.11 ± 1.23%; S237A + H23A: 5.86 ± 0.18%. The *p*-value of the one-way analysis of variance (ANOVA) test is 0.45 SD. In the box plots in subfigures (*B*), (*E*), and (*G*), the boundaries of the whiskers are based on an interquartile range of 1.5, and each *gray point* in the plot represents one measurement. *C*, overlay of averaged fluorescence response (25 periods) to 200-mV voltage steps from Helios WT and S237A, at 500 fps. *D*, S237A has fast kinetics as the upswing tau = 1.686 ms (fast component percentage 68.87%) and downswing tau = 1.849 ms (fast component percentage 80.62%). *E*, comparison between Helios WT and S237A rising fast time constants. Helios WT: 1.95± 0.45 ms (62%) (n = 3 cells); S237A: 1.72± 0.04 ms (82%) (n = 2 cells). The *p*-value of independent *t* test is 0.55. *F*, overlay of raw fluorescence response to a 200-mV voltage step from WT and S237A, at 500 fps. *G*, signal to noise ratio (SNR) bar graphs of the WT (3.93 ± 1.3/500 Hz, n = 48 measurements) and S237A (6.93 ± 1.0/500 Hz, n = 48 measurements). The *p*-value of independent *t* test is 4.1e-22. The SNR from S237A is significantly higher (76.3%) than that from the WT. *H*, Both the WT and S237A show no photocurrent upon 532-nm laser illumination. All the data shown here were acquired under an illumination intensity of 87.6 mW/mm^2^. All statistics are mean ± SD.

Because of the increased fluorescence brightness of the S237A mutant and positive effect on photobleaching, we focused our investigations of voltage sensitivity on S237A and mutant combinations with it (Fig. 4). We assessed the voltage sensitivity of S237A, S237A + H80A, and S237A + H23A (Fig. 4*A*). Sensitivities were (as ΔF/F per 200 mV): 6.14% ± 1.35% for Helios WT (n = 7 cells; all statistics are mean ± SD), 6.49% ± 1.4% for S237A (n = 4 cells), 5.11% ± 1.23% for S237A + H80A (n = 5 cells), and 5.86% ± 0.17% for S237A + H23A (n = 4 cells) (Fig. 4*B*). No statistically significant difference in voltage sensitivity was measured between any of the mutants. We compared the response speed of the S237A mutant to a 200-mV voltage step with that of Helios WT (Fig. 4*C*). We found that S237A had a response time of 1.69± 0.04 ms (82%) (n = 2 cells) and 1.95± 0.11 (86%) for the up- and downswing, respectively (Fig. 4*D*). We found no significant difference in the speed of voltage response between S237A and WT (Fig. 4*E*).

Given the relatively minor increase in brightness of S237A compared with WT and the similar voltage sensitivity and response speed, we were intrigued by the fact that the S237A fluorescence traces were substantially less noisy than the WT traces (Fig. 4*F*). We quantified the SNR with which we could detect a voltage step with Helios WT and S237A. We calculated the signal as the difference in the average fluorescence value for the 100 ms where the voltage was +100 mV, with the average fluorescence value for the 100 ms where the voltage was −100 mV. We calculated the noise as the standard deviation of the fluorescence for the 100 ms where the voltage was +100 mV. We found that the voltage detection SNR for S237A (6.93 ± 1.0/500 Hz, n = 48 measurements) is 76.3% higher than that of WT Helios (3.93 ± 1.3/500 Hz, n = 48 measurements). We wondered whether the increased noise in the WT recordings was due to photocurrent effects but measured no discernible photocurrent at −30 mV upon illumination with green light (532 nm, 87.6 mW/mm2) in either WT or S237A (Fig. 4*H*).

### Comparison of voltage-sensitive fluorescence between Arch and Helios

Voltage-sensitive fluorescence in Arch was shown to arise from photoexcitation of a prefluorescent 13-*cis* N-like state to a highly fluorescent Q-intermediate, either directly during the normal photocycle as in Arch WT (12) or due to photoexcitation of an accumulated all-*trans* O-intermediate as in Arch (D95N) (6). Early work on Bacteriorhodopsin also showed pH-sensitive fluorescence arising from excitation of the all-*trans* O-intermediate (30). The seconds-long photocycle (17) and short excited state lifetime (subpicoseconds) (31) of Helios suggests that we also measure photointermediate fluorescence under our imaging conditions. This could involve an accumulated O-intermediate, but probably *via* a different route due to its distinct photocycle. Contrary to Arch, Helios displays a long-lived *13-cis* O-intermediate, which reisomerizes to the ground state in seconds (17, 32). The preceding transition from the M to O state involves proton back-transfer from the PAG and reprotonation of the Schiff base, which could be influenced by membrane voltage. Further biophysical characterization of Helios will shed light on the photointermediates and transitions involved in its voltage-sensitive fluorescence.

Prior work on Arch variants indicates that the increase in fluorescence quantum yield arises from the protonated Schiff base and a neutral counterion, where voltage regulates the equilibrium between protonated and deprotonated SB (12, 33). This is influenced by the electrostatics of the RPSB environment and accessibility to proton transfer *via* the hydrogen-bonded network, which is quite different between Helios and Arch. The Arch binding pocket contains three water molecules (34), while for Helios at least seven internal water molecules have been reported in the retinal cavity (35). Furthermore, the primary proton transfer event from the RPSB occurs in the cytoplasmic direction in Helios, as opposed to the extracellular transfer in Arch. The back-transfer from the PAG in Helios could reduce the fidelity of Schiff base deprotonation under negative voltage, thereby limiting the voltage-sensitive response. These differences complicate a direct comparison between Arch and Helios. Nonetheless, we attempt to shed light on the discrepancy between their voltage sensitivities and influence of the counterion using insights from their crystal structures and mutation studies.

Neutralizing the Arch counterion (D95N/Q) leads to protonation and an increase in fluorescence under positive membrane voltage, which is not the case for the Helios counterion E107. In Helios, E107 does not stabilize the protonated SB as effectively as D95 in Arch. The weaker counterion interaction is compensated for by surrogate counterions involving other residues in combination with the water cavity and possibly anions. In addition, Arch has a second negatively charged counterion at D222, which is occupied by S237 in Helios. The large redshift of the S237A mutant indicates that this residue (in combination with the surrounding water network) is probably crucial in stabilizing the charge on the protonated SB (25). In the E107Q mutant, reorganization of the binding pocket stabilizes the RPSB due to interactions with S237 (36) or even anions, as E107Q can bind anions even at physiological pH values (37, 38). Thus, neutralizing the counterion in Helios (as in the E107Q mutant) is not analogous to the Arch D95N/Q mutants. However, our results with S237A demonstrate that this may be a useful fluorescence and/or sensitivity tuning site instead of E107, also due to evidence of its reorientation during proton transfer (24). A combination of mutations at S237 and other binding-pocket residues, which can stabilize the retinal protonated Schiff base, will likely improve the voltage-sensitive fluorescence of Helios.

## Conclusion

We investigated the potential of Heliorhodopsin as a GEVI and the effect of several mutations on its brightness, voltage sensitivity, photobleaching statistics, response speed, and photocurrent characteristics. The S237A mutant had a beneficial effect on fluorescence brightness without compromising photobleaching, voltage sensitivity, or response speed and can be used as a template for further protein evolution. Since S237A is directly hydrogen bonded to the RPSB near H80 and is an important color tuning residue, saturation mutagenesis of S237 or further mutant combinations in the binding pocket will likely yield improved variants. In addition, membrane-targeted expression of Helios variants can be improved by modifying the design of the expression construct, for instance, by rearranging of trafficking motifs, using a different fusion protein or inserting spacer elements.

We expect that future electrophysiological investigations into Heliorhodopsins might increase our understanding of its native function and exact photodynamics, which will aid further bioengineering efforts.

## Experimental procedures

### Plasmids and materials

The pBAD vector for recombinant expression of Heliorhodopsin HeR-48C12 containing an N-terminal 6xHis tag (pBAD-Helios-NT-6xHis) was a kind gift from Alina Pushkarev and Oded Béjà. The sequences for targeting and endoplasmic reticulum export motifs (TSX3ER2) and Citrine were derived from MPC020: CamKII CMV_NovArch_citrine, which was a gift from Adam Cohen (Addgene plasmid # 153193) (8). The pCAG backbone was derived from pCAG-Archon1-KGC-EGFP-ER2-WPRE, which was a gift from Edward Boyden (Addgene plasmid # 108423) (9).

### *E. coli* culturing and purification

pBAD-Helios-NT-6xHis was transformed into chemically competent *E. coli* cells (NEB 5-alpha, NEB) as per the manufacturer’s protocol. Overnight cultures were grown in LB medium under ampicillin selection (100 μg/ml) in a shaking incubator at 37 °C, 150 rpm. On the following day, the culture was diluted 1:50 times to a volume of 400 ml. Opsin expression was induced at *A*_600_ of 0.4 to 0.6 by adding a final concentration of 0.2% arabinose. All-*trans* retinal, 20 μM, dissolved in ethanol was added to the culture, and it was left shaking for another 14 to 18 h. The cells were pelleted by centrifugation at room temperature (RT), 4000*g*, 20 min and washed twice with an equal culture volume of 150 mM NaCl. The cell pellet was resuspended in 4 ml lysis buffer containing 50 mM Tris, 300 mM NaCl, 0.1% DDM and lysed using a French press. Membrane vesicles were pelleted by ultracentrifugation at 100,000*g*, 45 min, 4 °C. The pellet was resuspended in 50 mM Tris, 20 mM imidazole, 300 mM NaCl, 2% DDM, pH 6.5 and was left to mix for 1 h, RT. Insoluble debris was spun down at 100,000*g*, 45 min, 4 °C, and the supernatant was loaded onto a column containing Ni2+NTA resin for purification of the His-tagged protein using affinity chromatography. The resin was washed with 10 bed volumes of 50 mM Tris, 50 mM imidazole, 300 mM NaCl, 0.1% DDM, pH 6. The purified protein was eluted in 50 mM Tris, 500 mM imidazole, 300 mM NaCl, 0.1% DDM, pH 6 and concentrated using a 10 kDa spin column (Millipore). The absorption and emission spectra of the purified protein were recorded at RT (Lambda365, PerkinElmer and FLS980, Edinburgh Instruments).

### Confocal imaging

*E. coli* cells expressing pBAD-Helios-NT-6xHis were grown as described above. The cells were spun down, and the cell pellet was washed thrice and resuspended in an equal culture volume of PBS. The cell suspension was plated onto 35-mm imaging dishes with a 10-mm glass coverslip (Cellvis) coated with poly-L-lysine (Thermo Fisher). Fluorescence images were captured using laser-scanning confocal microscopy (Nikon Eclipse Ti inverted) at excitation wavelengths of 561 and 640 nm and emission at 595/50 nm and spinning disk (IX81, Olympus) confocal microscopy.

### Cloning

HeR-48C12 was amplified from pBAD-Helios-NT-6xHis and combined with TSX3ER2 and Citrine using overlap-extension PCR with Phusion high fidelity master mix (NEB). The primers used for cloning are listed in Table S1. TX3ER2 and Citrine were inserted at the C-terminal end of the protein, due to the inverted orientation of Heliorhodopsin in the membrane. The pCAG backbone was amplified using a high fidelity polymerase KODextreme hot start (Merck Sigma). TSX3ER2-Citrine-Helios was inserted into pCAG using Gibson assembly (Gibson assembly mastermix, NEB) to generate pCAG-Helios. Point mutations were generated by PCR using end-to-end primers with the mutation site encoded in the forward primer. KODextreme polymerase was used for amplification, and the product was ligated (KLD enzyme mix, NEB) and transformed into NEB 5-alpha Competent cells. The primer sequences used for mutagenesis are provided in Table S1.

### HEK cell culturing

HEK293T cells were grown at 37 °C, 5 to 10% CO_2_ in Dulbecco’s modified Eagle’s medium supplemented with 10% fetal bovine serum and penicillin/streptomycin. The cells were transfected at a confluency of ∼80% with 600 to 1000 ng of plasmid and 6 μl of TransIT293T (Mirus) transfection reagent. Twenty-four hours after transfection, the cells were plated onto 35-mm imaging dishes containing a 10-mm glass coverslip (Cellvis) coated with fibronectin (Merck).

### Patch clamp electrophysiology

Whole cell voltage clamp recordings were performed at room temperature (25 °C) 48 to 72 h after transfection. The cells were rinsed with extracellular buffer containing 125 mM NaCl, 2.5 mM KCl, 15 mM Hepes, 1 mM CaCl_2_, 1 mM MgCl_2_, and 30 mM Glucose, pH 7.3, osmolarity adjusted to 310 mOsm. Micropipettes were pulled from borosilicate glass capillaries (World Precision Instruments, 1.5 mm OD, 0.84 mm ID) using Next Generation Micropipette Puller (Sutter Instrument, P-1000) to obtain a pipette resistance of 5 to 10 MΩ. The pipettes were filled with intracellular buffer containing 125 mM potassium gluconate, 8 mM NaCl, 0.1 mM CaCl_2_, 0.6 mM MgCl_2_, 1 mM EGTA, 10 mM Hepes, 4 mM Mg-ATP, and 0.4 Na-GTP, pH 7.3, osmolarity adjusted to 295 mOsm. The micropipettes were positioned using the Patchstar micromanipulator (Scientifica). All patch clamp data were acquired in voltage clamp mode using the AM Systems Model 2400 Patch Clamp Amplifier, using voltage steps ranging from −100 to 100 mV. Simultaneous fluorescence measurements were performed as described below.

### Fluorescence imaging

These experiments were performed using a home-built multimodal microscope with a patch clamp add on, the design of which has been described recently (39). Epifluorescence imaging was performed using two laser beams at 488 nm (OBIS 488 LX, Coherent) and 532 nm (MLL-III-532, CNI) focused onto the back aperture of a 25X objective (XLPLN25XWMP2, Olympus). Illumination power of 11.2 mW/mm^2^ and 37.23 mW/mm^2^ were used for brightness screening for 488 nm and 532 nm, respectively. For patch clamp characterization, the 532 nm intensity was 87.6 mW/mm^2^. The emission light was filtered using a multiband dichroic mirror (Di03-R405/488/532/635-t3-32x44, Semrock) and a 552 to 779.5 bandpass filter (FF01-731/137-25, Semrock). The images were acquired at a frame rate of 100 or 500 Hz using an sCMOS camera (ORCA Flash4.0 V3, Hamamatsu; 2048 x 2048 pixels, 6.5 μm pixel size). The voltage pulses, illumination, and camera recording were synchronized using the National Instruments DAQ (USB-6363). All software for controlling the hardware, image acquisition and analysis were custom written in Python (39).

### Data analysis

From the recorded camera video, a background region was manually selected. The averaged background trace from this region was calculated, then smoothed. At each frame, time-corresponding background value was subtracted. A maximum-likelihood pixel weighting algorithm was utilized to extract the fluorescence trace. The trace was then corrected for photobleaching by normalization against the biexponential fitting of itself. An averaged period was calculated from it. To measure the change of fluorescence in response to membrane voltage, $F_{bl}$ (baseline fluorescence) and $F_{ss}$ (steady-state fluorescence) values were computed from fluorescence at resting potential and during voltage step after reaching steady state, then the sensitivity ΔF/F was presented as $\left(F_{ss}\;{-F}_{bl}\right)/F_{bl}$. To show the protein dynamics, the upswing and downswing phases were segmented, then fitted to a biexponential function: $F\left(t\right)=A\times \left(C\times \exp^{\left(-t/t_{1}\right)}+\left(1-C\right)\times \exp^{\left(-t/t_{2}\right)}\right)$, in which A is a constant, C is the magnitude percentage between two single exponential functions, $t_{1}$ is the fast time constant, and $t_{2}$ is the slow time constant. Statistical comparisons between datasets were performed as indicated in the figure captions, but were generally performed using Tukey’s method with one-way ANOVA tests. All data are means ± SD.

## Data Availability

Data underlying this publication have been deposited in the 4TU.REsearchData repository and are available under https://doi.org/10.4121/21401808.

## Supporting information

This article contains supporting information.

## Conflict of interest

The authors declare that they have no conflicts of interest with the contents of this article.
