# Supplementary material for: Expanding the family of genetically encoded voltage indicators with a candidate Heliorhodopsin exhibiting near-infrared fluorescence
Source: J Biol Chem. 2023 Apr 29;299(6):104771. doi: 10.1016/j.jbc.2023.104771 (PMC10238734; doi:10.1016/j.jbc.2023.104771)
Supplement: Supporting Figures S1–S3 and Tables S1 [file mmc1.docx]

**Supporting Information**

Expanding the family of genetically encoded voltage indicators with a candidate heliorhodopsin exhibiting near-infrared fluorescence

Srividya Ganapathy^1,2^*, Xin Meng^1^*, Delizzia Mossel^1^, Mels Jagt^1^, Daan Brinks^1,3^

^1^Department of Imaging Physics, Delft University of Technology, Delft, The Netherlands

^2^Department of Pediatrics & Cellular and Molecular Medicine, UCSD School of Medicine, San Diego, USA

^3^Department of Molecular Genetics, Erasmus University Medical Center, Rotterdam, The Netherlands

*these authors contributed equally to this work

**Table S1**

| **Primer name** | **Sequence** |
| --- | --- |
| Helios-fwd | ctgtacaaaAAGGAGATATACATACCCATGG |
| Helios-rev | aagcttgatatcgaattcGTTTAAACTTAAGCAGGAATTAAC |
| TSX3ER2-fwd | ttctgggggcagcgggggatccaccATGTCAAGGATCACCTCTG |
| TSX3ER2-rev | cgcccttgctcacTTCATTCTCATAACAAAAGCC |
| Citrine-fwd | tatctccttTTTGTACAGCTCGTCCATG |
| Citrine-rev | tatctccttTTTGTACAGCTCGTCCATG |
| FCK-fwd | GAATTCGATATCAAGCTTATC |
| FCK-rev | GGTGGATCCCCCGCTGCC |
| pCAG-fwd | TGTACAAGTAAAGCGGCCC |
| pCAG-rev | AACCATGGTGGCGGTACC |
| TSX3ER2-Citrine-Helios-fwd | accgccaccatggttTCAAGGATCACCTCTGAGG |
| TSX3ER2-Citrine-Helios-rev | ggccgctttacttgtacaTTAAGCAGGAATTAACGTTCC |
| Helios-E107N-fwd | tcgttgggttAACtattcactttcttc |
| Helios-E107Q-fwd | tcgttgggttCAGtattcactttcttc |
| Helios-E107-rev | AAGATGTTTTGATTCTTCATAAG |
| Helios-H80A-fwd | tctgctctttttGCCtttattgtttc |
| Helios-H80-rev | caacccaagaaatagcgcaactg |
| Helios-S237A-fwd | tacattgtgttgGCCttagttgcaaaatc |
| Helios-S237-rev | agcacgttctcctctgaggtaatta |
| Helios-H23A-fwd | gctggagtttttGCCcttttgcaaatg |
| Helios-H23-rev | aattctgttgaagttttgtaagga |

**Table S1.** **Primer sequences used in this study.** Primers used for Gibson assembly cloning of Helios into the expression construct used in this study and for further generating the various site-directed mutants described here.

**Figure S1**


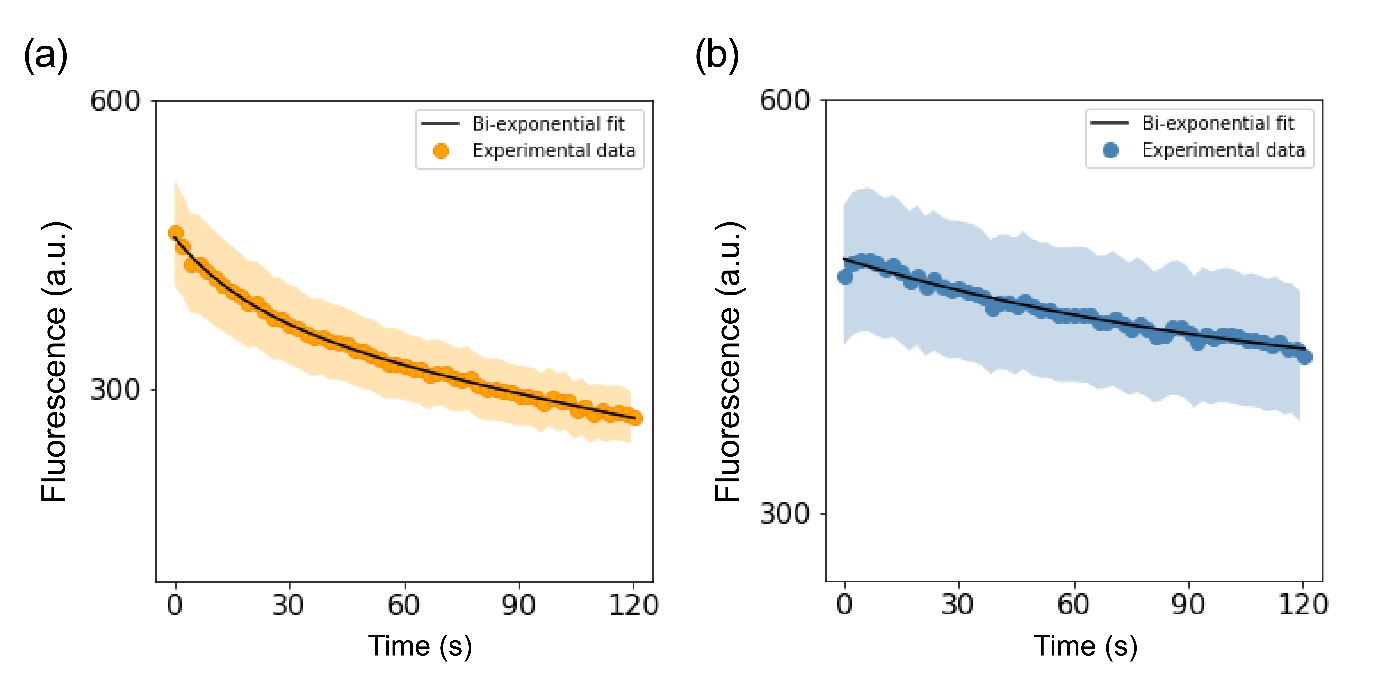


**Fig. S1. Photobleaching of Helios expressed in *E. coli*.** Photobleaching curves of Helios under 561 nm (nm) and 640 nm (b) laser-scanning confocal illumination acquired at a scan speed of 1 fps.

**Figure S2**


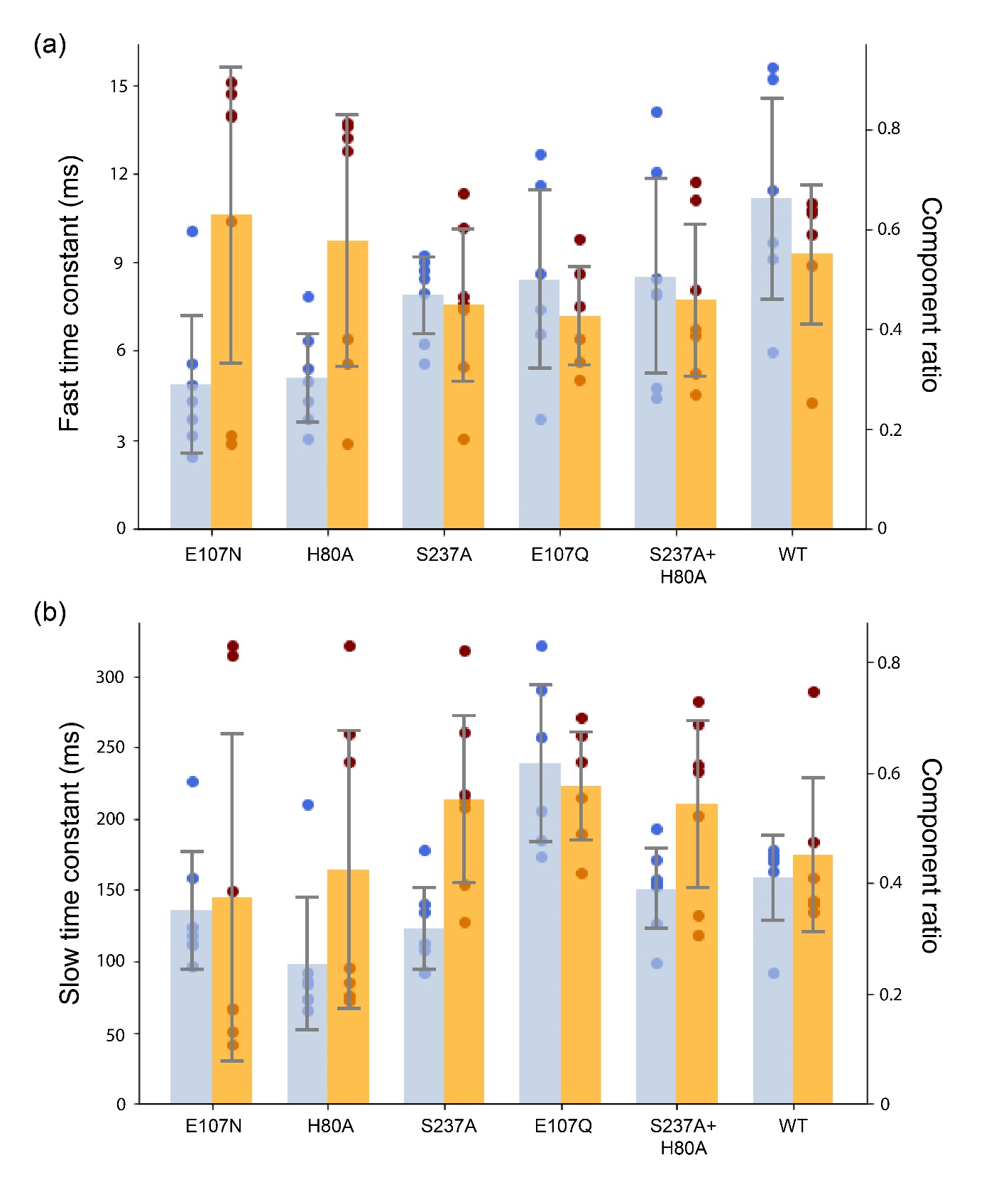


**Fig. S2. Photobleaching timeconstants and component percentages for 6 Helios mutants**. Result bioexponential fits to the photobleaching curves recorded under 87.6 mW/mm2 illumination with 532 nm.

**Figure S3**

**
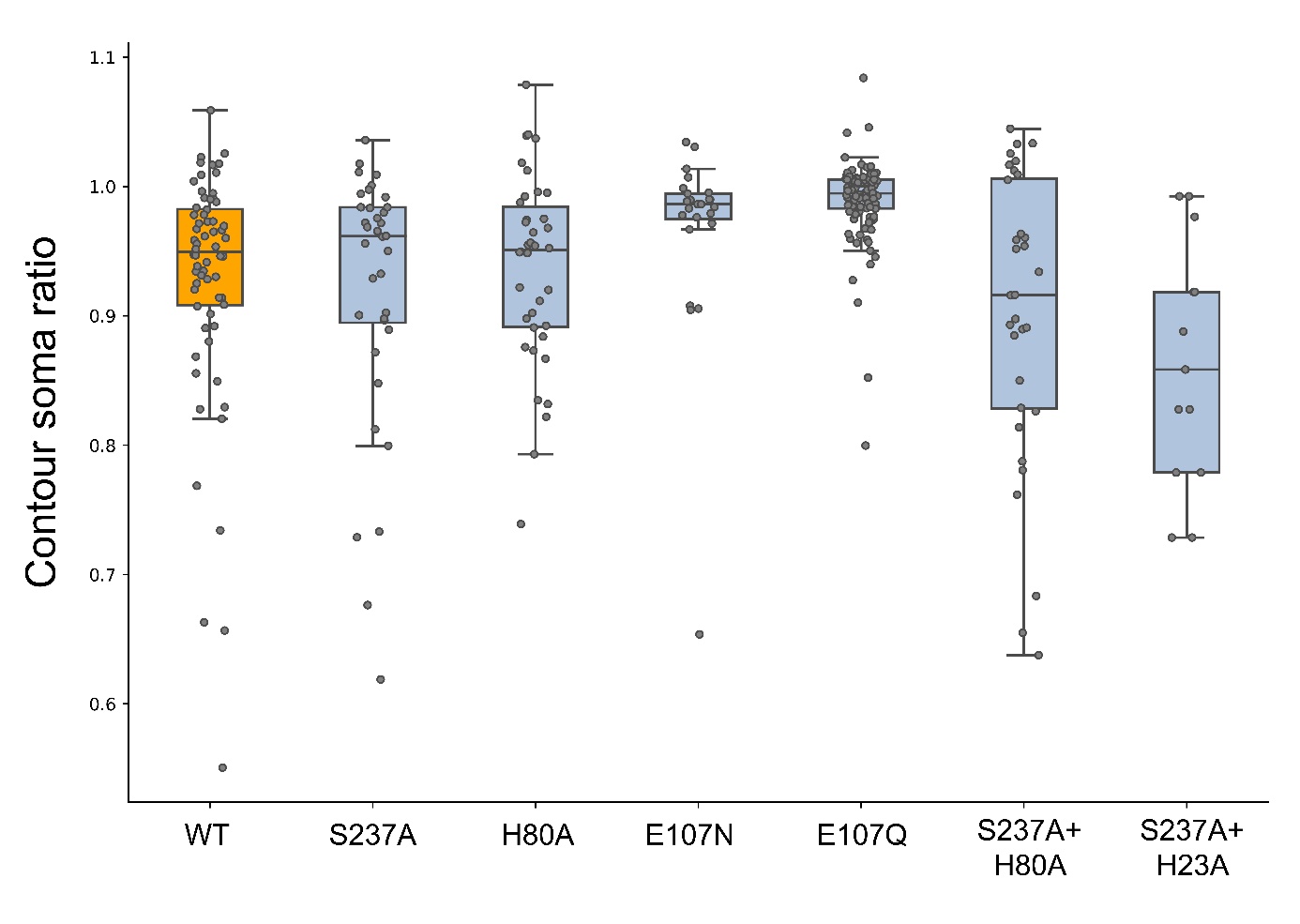
**

**Fig. S3.** **The fluorescence ratios between the cell contour membrane and soma expression from Helios WT and its mutants**. The boundaries of the whiskers are based on an interquartile range of 1.5, and each grey point in the plot represents one measurement.
